# Specificity of HTLV screening tests and its impact on health care program costs: The perspective of antenatal screening in Brazil

**DOI:** 10.1590/0037-8682-0853-2020

**Published:** 2021-03-08

**Authors:** Carolina Rosadas, Adele Caterino-de-Araujo, Graham Philip Taylor

**Affiliations:** 1 Imperial College London, Section of Virology, Department of Infectious Disease, London, United Kingdom.; 2 Instituto Adolfo Lutz, Centro de Imunologia, Laboratório de Pesquisa em HTLV, São Paulo, SP, Brasil.; 3 Imperial College Healthcare NHS Trust, St. Mary’s Hospital, National Centre for Human Retrovirology, London, United Kingdom.

**Keywords:** Human T cell lymphotropic virus (HTLV), Diagnosis, Screening assays, Specificity, Antenatal care program, Cost-effectiveness

## Abstract

**INTRODUCTION:**

Brazil ranks first in the number of HTLV-1/-2-infected individuals worldwide. The high morbidity and mortality of HTLV-1-associated diseases, especially following infection in infancy, requires strong action to reduce vertical transmission.

**METHODS:**

To facilitate the appraisal of the implementation of the HTLV antenatal screening program by the Brazilian Ministry of Health, we determined the costs in distinct scenarios according to HTLV seroprevalence, specificity of the screening test, and type of confirmatory test.

**RESULTS:**

HTLV antenatal screening would cost R$ 55,777,012-R$ 77,082,123/year. Screening assays with high specificity reduce the need and cost of confirmatory assays by up to 25%.

**CONCLUSIONS:**

Careful selection of the screening assay is required to optimize the program.

Human T-cell lymphotropic virus 1 (HTLV-1) is a retrovirus, currently infecting at least 5-10 million individuals worldwide. In Brazil, the estimated number of people living with HTLV-1 is high (800,000-2.5 million)^1^ ^,^ ^2^. HTLV-1 is transmitted through unprotected sexual intercourse, exposure to infected blood and organs, and from mother to child mainly via breastfeeding. This virus is associated with a range of diseases, such as chronic myelopathy (HTLV-1 associated myelopathy) and severe leukemia (adult T cell leukemia), both of which are well characterized. The deleterious impact of HTLV-1 in other infections, such as *Strongyloides stercoralis* and *Mycobacterium tuberculosis*, is evident in many countries, including Brazil, and constitutes a public health problem^3^. Most people living with HTLV remain asymptomatic throughout their lifetime. However, the reduced quality of life due to HTLV-1 infection is becoming more evident, even in those considered asymptomatic^4^.

The diagnosis of HTLV-1/2 infection is based on the detection of anti-HTLV-1/2 antibodies using a screening assay, such as enzyme-linked immunoassay (ELISA) or chemiluminescent immunoassay (CMIA), followed by confirmatory testing of the reactive samples. There is a range of commercially available assay kits for the initial detection of HTLV-1 antibodies, and their performances are diverse with specificities varying from 92.0% to 99.5% in the Brazilian setting^5^. Western blotting (WB) and line immunoassay (LIA) are used to confirm the presence of specific antibodies to HTLV-1/2, while in-house polymerase chain reaction (PCR) assays can confirm the presence of HTLV proviral DNA in peripheral blood mononuclear cells (PBMC)^6^ ^,^ ^7^. Confirmatory tests are also used to distinguish HTLV-1 from HTLV-2^6^. This differentiation is important, as HTLV-2 is rarely associated with disease.

The identification of seropositive individuals is crucial to prevent new infections, but policymakers may consider the cost of testing as a limiting factor. Few studies have focused on reducing the cost of HTLV testing. 

Here, we analyzed the impact of the performances of HTLV-1 screening tests on healthcare expenditure in the context of a national antenatal screening program in Brazil. Additionally, we determined the economic impact of national antenatal screening in the country, using the perspective of the Brazilian Health Unified System (Sitema Único de Saúde, SUS). For this purpose, we considered the need for a diagnostic algorithm comprising one screening test (ELISA/CMIA), followed by confirmatory test (either WB or PCR) on seropositive samples. 

The following inputs were considered:

1. Number of pregnant women in Brazil in 2018 (extrapolated from the number of live births in Brazil): 2,944,932 (TABNET, applicative that allows DATASUS consultation, Ministério da Saúde do Brasil)
2. HTLV-1 screening test specificities: 92.0% (Murex HTLV-1/2), 98.1% (Architect rHTLV-1/2), 99.0% (Anti-HTLV-1/2 SYM Solution), and 99.5% (Gold ELISA)^5^
3. HTLV-1 screening test sensitivity was 100% for each of the above assays
4. Prevalence of HTLV-1 infection in pregnant women in Brazil (Minimum 0.1%-Maximum 1.05%)^12^
5. Cost of ELISA: R$ 18.55 according to price list management of SUS (SIGTAP, Ministério da Saúde do Brasil, December 2020)
6. Cost of western blot: R$ 85.00 (SIGTAP, Ministério da Saúde do Brasil, December 2020)
7. Cost of PCR: R$ 65.00 (SIGTAP, Ministério da Saúde do Brasil, December 2020)

The Brazilian Ministry of Health will have to spend R$ 54,628,489/year on initial screening for HTLV using ELISA, based on the number of pregnant women in Brazil and the cost of ELISA, as defined by SUS. 

The number of false positive results was calculated by multiplying the total number of pregnant women with (1 − Specificity) × (1 − Prevalence). Next, the cost of testing to confirm and discriminate HTLV-1 and HTLV-2 infections was determined for both WB and PCR.

The confirmation of HTLV-1/2 infection is strongly recommended, and the cost will vary according to the specificity of the screening test, the prevalence of HTLV-1 infection, and the choice of the assay, as detailed in Table 1. Briefly, the Brazilian Ministry of Health would spend on antenatal screening about R$ 56,130,404-R$ 77,082,123 if using WB and about R$ 55,777,012-R$ 71,798,915 if PCR is used as the confirmatory assay; however, if using PCR, the women with reactive serological samples would need to be re-called and a new blood sample collected, as PCR is performed using PBMCs (Table 1). 

**TABLE 1:** Estimation of economic impact for the implementation of HTLV-1/2 antenatal screening in Brazil and its variation according to HTLV prevalence, assay specificity, and type of confirmatory test.

Assay specificity	Positive in HTLV screening test			Cost of confirmatory assay^a^		Total cost of antenatal screening^a^	
	HTLV-1 infected (expected n)	False positive (expected n)	Total positive screening (n)	WB (R$)	PCR (R$)	ELISA + WB (R$)	ELISA + PCR (R$)
**0.1% HTLV prevalence**							
92.0%	2,945	235,300	238,245	20,250,825	15,485,925	74,879,313	70,114,414
98.1%	2,945	55,954	58,899	5,006,384	3,828,412	59,634,873	58,456,900
99.0%	2,945	29,449	32,394	2,753,511	2,105,626	57,382,000	56,734,115
99.5%	2,945	14,725	17,670	1,501,915	1,148,523	56,130,404	55,777,012
**1.05% HTLV prevalence**							
92.0%	30,922	233,239	264,160	22,453,634	17,170,426	77,082,123	71,798,915
98.1%	30,922	55,365	86,287	7,334,353	5,608,623	61,962,842	60,237,112
99.0%	30,922	26,504	57,426	4,881,225	3,732,701	59,509,713	58,361,190
99.5%	30,922	14,430	45,352	3,854,916	2,947,877	58,483,405	57,576,366

^a^Cost in brazilian reais (R$) according to the price list management of SUS (SIGTAP), Ministério da Saúde do Brasil. Based on 2,944,932 pregnant women.

Therefore, using a screening test with higher specificity could result in a reduced cost of up to 25.0% (19.8%-25.0%), saving up to R$ 18,748,909/year (Table 2). This would also result in up to 220,575 fewer false-positive results.

**TABLE 2:** Maximum savings and cost minimization achievable on HTLV-1/2 antenatal screening according to the performance of the screening test.

	Maximum savings^a^ (R$)		Cost minimization^a^ (%)	
	ELISA + WB	ELISA + PCR	ELISA + WB	ELISA + PCR
0.1% prevalence	18,748,909	14,337,402	25.0	20.4
1.05% prevalence	18,598,718	14,222,549	24.1	19.8

^a^ Cost in brazilian reais (R$) according to the price list management of SUS (SIGTAP), Ministério da Saúde do Brasil.

Additionally, the diagnosis of HTLV-1 infection during pregnancy is extremely important because this route of infection is associated with a higher risk of HTLV-1 associated diseases. The identification of infected pregnant women represents a unique opportunity to block the silent transmission of HTLV within families and communities, as it allows the implementation of effective measures to significantly reduce the HTLV-1 mother-to-child transmission. The Brazilian Ministry of Health recommends to avoid breastfeeding for all HTLV-1/2 seropositive mothers and provides formula for feeding^13^. However, antenatal screening is not implemented throughout the country, being performed only in some specific states, such as Bahia, due to independent local policies. 

LIA has superior performance than WB, giving fewer indeterminate results^6^ ^,^ ^14^ ^,^ ^15^. In this study, we did not consider this assay, as it is not included in the range of tests offered by SUS for the diagnosis of HTLV-1/2 infection. Based on increasing evidence, the Brazilian public health system should consider including LIA as an option for confirmatory testing for HTLV-1/2 infection, as is the case in Japan^7^. However, the costs must be discussed, as LIA is more expensive than WB.

Our analysis focused on individual sample testing. However, the use of pooled sera for HTLV-1/2 screening is an acceptable strategy to reduce cost^8^ ^,^ ^9^ and was part of the screening protocol for blood donors in the United Kingdom between 2002 and 2013^10^. A recent study confirmed that the use of pooled sera for HTLV-1 screening did not affect the diagnostic sensitivity and specificity of ELISA screening and could reduce cost up to 73.6% when applied to samples of at-risk populations in Brazil^11^. The introduction of pooling would also require considerable changes to laboratory processes, but these might be considered if they are applicable to other screening tests.

In summary, we have demonstrated that, regardless of the cost of the screening assay for the Brazilian Ministry of Health, using an assay with high specificity would allow a significant reduction in overall expenditure. The best performing screening assay would not only result in economic savings but would also prevent distress and uncertainty in up to 220,575 pregnant women per year, who would no longer receive a false-positive result in the screening test (235,300-14,725 in a scenario with 0.1% HTLV prevalence). This also reinforces the importance of immediate confirmatory testing for assays with low specificity, as it is unacceptable to misinform up to 80 women regarding HTLV-1/2 seropositivity for every true positive infection detected (up to 235,300 false positive/2,945 true positive). We also determined the cost of the implementation of HTLV-1/2 national antenatal screening in Brazil, which will facilitate the appraisal of this important public policy that is necessary in Brazil.
